# Effects of Hip Bracing on Gait Biomechanics, Pain and Function in Subjects With Mild to Moderate Hip Osteoarthritis

**DOI:** 10.3389/fbioe.2022.888775

**Published:** 2022-07-11

**Authors:** Hannah Steingrebe, Bernd J. Stetter, Stefan Sell, Thorsten Stein

**Affiliations:** ^1^ BioMotion Center, Institute of Sports and Sports Science, Karlsruhe Institute of Technology (KIT), Karlsruhe, Germany; ^2^ Sports Orthopedics, Institute of Sports and Sports Science, Karlsruhe Institute of Technology (KIT), Karlsruhe, Germany; ^3^ Joint Center Black Forest, Hospital Neuenbürg, Neuenbürg, Germany

**Keywords:** osteoarthritis, bracing, walking, gait analysis, pain, functional capacity, proprioception, conservative treatment/methods

## Abstract

Hip Osteoarthritis (HOA) is a common joint disease with serious impact on the quality of life of the affected persons. Additionally, persons with HOA often show alterations in gait biomechanics. Developing effective conservative treatment strategies is of paramount importance, as joint replacement is only indicated for end-stage HOA. In contrast to knee osteoarthritis, little is known about the effectiveness of hip bracing for the management of HOA. Studies analysing mechanically unloading hip braces partly showed beneficial results. However, methodological limitations of these studies, such as small sample sizes or lack of control groups, limit the applicability of the results. Additionally, mechanically unloading braces might impose restrictions on motion and comfort and thus, might not be suitable for people with only mild or moderate symptoms. The aim of this study was to comprehensively quantify the effects of unilateral HOA as well as functional hip bracing on gait biomechanics, pain, proprioception and functional capacity in people with mild to moderate HOA. Hip and pelvis biomechanics during walking were analysed in 21 subjects with mild to moderate HOA under three bracing conditions: unbraced, immediately after brace application and after 1 week of brace usage. Additionally, pain, hip proprioception and functional capacity were assessed. A matched group of 21 healthy subjects was included as reference. Kinematic and kinetic data were collected using a 16-camera infrared motion capturing system and two force plates. Visual analogue scales, an angle reproduction test and a 6-min walking test were applied to measure pain, hip proprioception and functional capacity, respectively. Subjects with HOA walked slower, with reduced step length, sagittal hip range of motion and peak extension angle and had a reduced functional capacity. After 1 week of brace application step length, walking speed and functional capacity were significantly increased. Additionally, pain perception was significantly lower in the intervention period. These results encourage the application of functional hip braces in the management of mild to moderate HOA. However, as key parameters of HOA gait such as a reduced peak extension angle remained unchanged, the underlying mechanisms remain partly unclear and have to be considered in the future.

## 1 Introduction

Osteoarthritis (OA) is a degenerative joint disease with the hip joint being one of the most commonly affected joints (Litwic et al., 2013; Turkiewicz et al., 2014). The risk of developing hip OA (HOA) increases with age (Bijlsma and Knahr, 2007): about 28% of people above the age of 45 show radiographic signs of HOA and 9.7% develop symptomatic HOA (Jordan et al., 2009). Due to demographic changes, the number of people suffering from HOA is likely to increase in the future (Fuchs et al., 2017). This stresses the need for effective treatment strategies as the emergence of HOA has serious consequences for the quality of life of the affected persons (Salaffi et al., 2005).

Previous studies have shown that people suffering from various degrees of HOA show altered gait kinematics including a reduced walking speed, step length and cadence (Hurwitz et al., 1997; Hulet et al., 2000; Watelain et al., 2001; Kubota et al., 2007; Ornetti et al., 2010; Eitzen et al., 2012; Constantinou et al., 2017). Additionally, a reduced sagittal plane range of motion (ROM) as well as peak extension angle have been observed (Altman et al., 1991; Hurwitz et al., 1997; Watelain et al., 2001; Pua et al., 2009; Eitzen et al., 2012; Kumar et al., 2015; Constantinou et al., 2017) accompanied by a discontinuity of the hip extension movement (Hurwitz et al., 1997; Foucher et al., 2012). Frontal and transverse plane hip kinematics have been less frequently analysed but some studies reported reduced hip adduction angles (Tateuchi et al., 2014; Leigh et al., 2016; Meyer et al., 2018) and decreased hip internal rotation (Leigh et al., 2016). Furthermore, changes in pelvic kinematics have been found and interpreted as compensatory mechanisms for limited hip mobility (Murray et al., 1971; Watelain et al., 2001). In the sagittal plane, Murray et al. (1971), Kubota et al. (2007) and Leigh et al. (2016) showed an increased anterior tilt while Watelain et al. (2001) reported increased posterior tilt at push-off as well as an increased ROM. In the frontal plane, again an increased ROM was found as well as an increased pelvis drop on the unsupported side (Watelain et al., 2001). Additionally, Murray et al. (1971), Thurston (1985), and Leigh et al. (2016) reported increased transverse pelvic rotation.

Hip joint loading studied by measuring joint moments revealed reduced external hip flexion and hip extension moments (Hurwitz et al., 1997; Watelain et al., 2001; Eitzen et al., 2012; Tateuchi et al., 2014; Foucher, 2017), reduced external hip adduction (Hurwitz et al., 1997; Foucher, 2017) and abduction moments (Kubota et al., 2007; Meyer et al., 2018) as well as reduced internal and external rotation moments (Hurwitz et al., 1997) for people with HOA. A recent meta-analysis highlighted that these reductions of joint moments cannot be solely attributed to a reduced walking speed but rather are the result of altered movement patterns (Diamond et al., 2018). Thereby, the reduction of joint moments seems to depend on HOA severity with a reduction present in people with end-stage HOA but not in people with moderate HOA (Diamond et al., 2018).

In addition to gait impairments, several studies have shown impairments in postural control in people with HOA (Truszczyńska et al., 2016; Picorelli et al., 2018; Slomka et al., 2019), which have partly been attributed to a loss of proprioception (Slomka et al., 2019). Proprioception strongly relies on the afferent information of a group of mechanoreceptors (Gardner, 2021), of which there is a significantly lower number in the hip joint capsule, labrum and femoral head ligament of people with HOA (Moraes et al., 2011). Decreased joint position sense as one measure of proprioceptive acuity has been frequently reported for knee osteoarthritis (KOA) patients (Barrett et al., 1991; Knoop et al., 2011) while, to our knowledge, no studies exist for HOA patients.

To date, it is still not clear whether these gait modifications solely reflect adaptions to pain and movement constraints or to some extent are provoking HOA and driving degeneration of the affected joint (Hurwitz et al., 1997; Eitzen et al., 2012; Leigh et al., 2016). However, persistent gait modifications might cause subsequent muscle weaknesses in less used muscles (Hurwitz et al., 1997) causing a downward spiral of poor posture, pain and decrease of functional capacity. Therefore, treatment strategies should aim at pain relief and normalization of the gait pattern to avoid secondary damage to adjoining soft tissue areas and joints (Hurwitz et al., 1997; van Drongelen et al., 2020) Additionally, reduction of disease progression and increase of functional capacity should be strived to enhance the perceived quality of life (Perrot, 2012). Overall, conservative treatment strategies, such as exercise, are of paramount importance for the management of symptoms, as joint replacement is only indicated for end-stage OA (Bennell and Hinman, 2011; Murphy et al., 2016). However, in contrast to KOA, profound knowledge about conservative, non-pharmacological treatments for HOA is still sparse and current guidelines for HOA treatment often solely recommend exercise due to a lack of reliable data on other conservative treatment options (Bennell and Hinman, 2011). One aspect of conservative treatment strategies is the use of braces. While brace usage has been extensively studied (Beaudreuil et al., 2009; Duivenvoorden et al., 2015; Cudejko et al., 2018) and is often recommended for people with KOA (Kolasinski et al., 2020), only limited studies exist analysing their therapeutic effectiveness in people with HOA.

Most previously-published studies analysed the effects of braces aiming to mechanically unload the hip joint (Shiba et al., 1998; Sato et al., 2008, 2012; Yamaji et al., 2009; Nérot and Nicholls, 2017). Of these, the first (Shiba et al., 1998) studied the effects of the Hip Joint Moment Reduction Brace and showed that the brace was able to reduce hip abductor muscle activity during walking by 32.6%. However, these results were observed only in a small population of healthy subjects and no inverse dynamic analysis of hip moments was conducted. Additionally, the authors stated that use of such an unloading brace might impact the ability to conduct activities of daily living which limits the area of application. Next, Sato and colleagues analysed the effects of the WISH brace designed to restrict hip adduction and exert pressure on the greater trochanter. Brace wear resulted in pain relief and reduced the dependency on analgesics (Sato et al., 2008, 2012); however, due to a simultaneous walking exercise therapy the effects might not be solely attributable to brace application. Yamaji et al. (2009) analysed the effects of the WISH brace on ground reaction forces (GRF) during gait and found an increase in the first peak of the vertical GRF. However, a very small sample size of seven subjects was studied and no joint kinematics or kinetics were reported, limiting the insights of these results. Another brace for subjects with HOA aims to reduce the internal hip abduction moment by externally applying hip abduction and external rotation forces (Nérot and Nicholls, 2017). Brace application resulted in a significant reduction of the peak adduction and internal rotation angles as well as the peak internal abduction moment during the stance phase of level walking. Yet, the effects on pain perception varied strongly between subjects (Nérot and Nicholls, 2017). Additionally, only immediate brace effects have been studied and data of the HOA subjects were not compared to a healthy control group (CG) to analyse normalization of movement patterns due to brace application.

Although some of the analysed braces showed positive results on (e.g.) pain perception, hip braces are currently nearly exclusively used after hip arthroplasty to prevent excessive joint motion (Yonclas et al., 2006). Beside the generally sparse availability of studies, one reason for this might be the discomfort experienced by patients due to the weight and stiffness of hip braces designed to mechanically stabilize and unload the affected hip joint. Additionally, rigid braces that restrict motion might not be suitable for a population with moderate symptoms still engaging in sports or daily activities (Shiba et al., 1998).

Modern definitions of OA stress that it does not exclusively involve the joint cartilage but affects all joint structures (Block and Shakoor, 2009). Additionally, it has been shown for HOA that the correlation between symptoms and radiographic signs is inconsistent (Kinds et al., 2011). Therefore, brace concepts focussing on soft tissue joint structures such as muscles or joint capsules might be beneficial for the patients without the necessity of hard shells to exert force on the hip joint. As stated previously, current guidelines for HOA treatment often recommend the application of exercise added by physiotherapeutic treatments such as manual therapy and massage (Hernández-Molina et al., 2008; Cibulka et al., 2017; The Royal Australian College of General Practitioners, 2018; Bannuru et al., 2019; Kolasinski et al., 2020). Thus, applying conservative treatments such as friction and trigger point massage by a brace might, due to the longer application period, be beneficial for pain and joint stiffness, and subsequently allow HOA patients to enhance their physical activity level. Additionally, the use of elastic bandages and braces has been shown to increase the proprioceptive capacity of healthy (Baltaci et al., 2011), KOA (Barrett et al., 1991; Birmingham et al., 2001) as well as ACL-deficient subjects (Birmingham et al., 2001b). However, results regarding the effectiveness for people with HOA are still lacking.

Therefore, the aims of the present study were first to analyse the effect of HOA on gait kinematics and dynamics, proprioception and functional capacity in a population with unilateral symptomatic HOA. Based on previous findings we hypothesized that subjects with HOA would show a 1) reduced walking speed and step length; 2) reduced sagittal plane ROM and peak hip extension angle; 3) decreased hip proprioception; and 4) decreased functional capacity compared to a healthy CG.

Secondly, we aimed to evaluate the short- and mid-term effects of hip brace application on gait kinematics and dynamics, pain, function and proprioception. We expected brace application to 5) reduce pain perception; 6) enhance hip proprioception; and 7) increase functional capacity. For the hip kinematics we expected 8) increased sagittal hip ROM and peak hip extension angle, resulting in 9) longer step length and faster walking speed. While we expected 10) short-term brace application to have an immediate effect on hip proprioception, we expected 11) the effects on functional capacity and gait biomechanics to arise only after mid-term brace application.

## 2 Materials and Methods

A case-control study and an intervention study were combined to investigate the research questions.

### 2.1 Subjects

In total, 42 subjects participated in this study. The intervention group (HOA group) was formed by 21 subjects with unilateral symptomatic moderate HOA. The sample was an ad hoc sample of convenience and was recruited through local physiotherapy practices as well as via university information events concerning OA care. Assessment of HOA was based on clinical as well as radiographic criteria, as this provides higher sensitivity and specificity (Altman et al., 1991). Evaluation of the radiological images and classification of radiological OA signs based on the Kellgren-Lawrence-Score (K-L-Score; Kellgren and Lawrence, 1957) was conducted by the same experienced orthopaedist.

The control group was formed by 21 healthy subjects without hip pain and was matched to fit the mean age, weight and height of the HOA group. Based on the involved side of the HOA group, 11 subjects were randomly assigned to the right hip group, 10 subjects to the left hip group.

The inclusion and exclusion criteria for the HOA and control groups are specified in Table 1. Details of the two subject groups are presented in Table 2.

**TABLE 1:** Inclusion and exclusion criteria for the HOA and control groups.

Inclusion criteria	Exclusion criteria
HOA group	
Radiologically confirmed HOA	Secondary HOA caused by trauma
- K-L-Score 2–4	
Hip pain within the last 3 months during activities of daily living	Neuromuscular disorders or neurological complaints (e.g. vertigo)
Decreased hip function	Contraindication of X-ray imaging
- Harris Hip Score 65–95	
Asymptomatic contralateral hip	BMI ≥35 kg/m^2^
- K-L-Score ≤ 2	
- no hip pain within the last 3 months	
- unrestricted passive range of motion (ROM)	Orthopaedic injury of other joints of the lower limbs and back (e.g. pain, osteoarthritis > grade 1 (self-reported), endoprosthesis, rheumatoid arthritis, acute herniated disc etc.)
- sagittal ROM ≥90°	
- transverse plane ROM ≥15°	
- peak abduction ≥20°	
- flexing contracture ≤10°	
Control group	
No radiological signs of HOA	Orthopaedic injury of other joints of the lower limbs and back (e.g. pain, osteoarthritis > grade 1 (self-reported), endoprosthesis, rheumatoid arthritis, acute herniated disc etc.)
- Bilateral K-L-Score ≤ 1 (if radiographic images available)	
No hip pain within the last 3 months during activities of daily living	Neuromuscular disorders or neurological complaints (e.g. vertigo)
Good hip function	BMI ≥35 kg/m^2^
- Harris Hip Score ≥96	

**TABLE 2:** Mean values and standard deviations of the subject characteristics of the HOA and control groups with respective *p*-values as revealed by independent sample t-tests/Mann-Whitney-U tests (MWU). Level of significance ≤0.05.

	HOA group (n = 21)	Control group (n = 21)	p (*t*-test/MWU)
Gender	11 male, 10 female	11 male, 10 female	
Age [years]	64.0 (9.6)	63.1 (9.2)	0.769
Body mass [kg]	71.3 (11.9)	74.4 (12.7)	0.429
Height [cm]	171.2 (6.7)	171.1 (8.8)	0.981
Body Mass Index (BMI) [kg/m^2^]	24.2 (2.9)	25.2 (2.7)	0.257
Harris Hip Score	74.6 (11.8)	98.4 (2.3)	<0.001*
Hip Osteoarthritis Outcome Score (HOOS)	62.0 (16.4)	97.7 (5.1)	<0.001*
Tegner Activity Score	4.7 (0.8)	4.9 (1.2)	0.370
Involved/analysed side	11 right, 10 left	11 right, 10 left	
K-L Score	Grade 2 = 9		
	Grade 3 = 7		
	Grade 3/4 = 1		
	Grade 4 = 4		

The study procedure was approved by the ethical committee of the Karlsruhe Institute of Technology. All participants gave their written informed consent prior to study participation.

### 2.2 Measurement Methods

To assess hip and pelvis biomechanics, three-dimensional gait analysis was conducted using a 16-camera infrared motion capturing system (Vicon, 200 Hz, Vicon Motion Systems, Oxford Metrics Group, Oxford, United Kingdom) and two 3D force plates (AMTI, 1,000 Hz, BP600900, Advanced Mechanical Technology Inc., Watertown, MA, United States). A full-body marker set with 42 retroreflective markers was used. Marker locations are specified in the Supplementary Material.

Sagittal plane hip proprioception was assessed using an active-active angle reproduction test. The testing procedure was based on the work of Arvin et al. (2015) with some structural changes to increase test feasibility and reliability (Steingrebe et al., 2019). Subjects stood upright on a block of 20 cm height allowing the ipsilateral leg to swing freely. Subjects were blindfolded to eliminate any visual information and held onto a horizontal bar to facilitate balance. After a “Start” command, subjects actively flexed their hip in a slow and steady manner until a “Stop” command was given by the instructor. When the subjects felt to have sufficiently memorized the adopted position they pressed a button, fixed at the handlebar, which powered an infrared light appearing in the simultaneous motion capture recordings and then returned to the initial position. After a rest period of 3 s another “Start” command was given and subjects replicated the previously-adopted position as precisely as possible, pushing the button when they felt they were in the correct position. A rest period of 30 s was given between each of the five trials.

As recommended by Cibulka et al. (2009), functional capacity was assessed using a 6-minute walking test (6MWT) (Rejeski et al., 1995; Enright, 2003) on a standardized circuit of 54 m length completed in a counter-clockwise manner. Subjects were asked to rate their current level of hip pain directly before and after completion of the test on a visual analogue scale of 10 cm length.

### 2.3 Study Procedure

**FIGURE 1:**
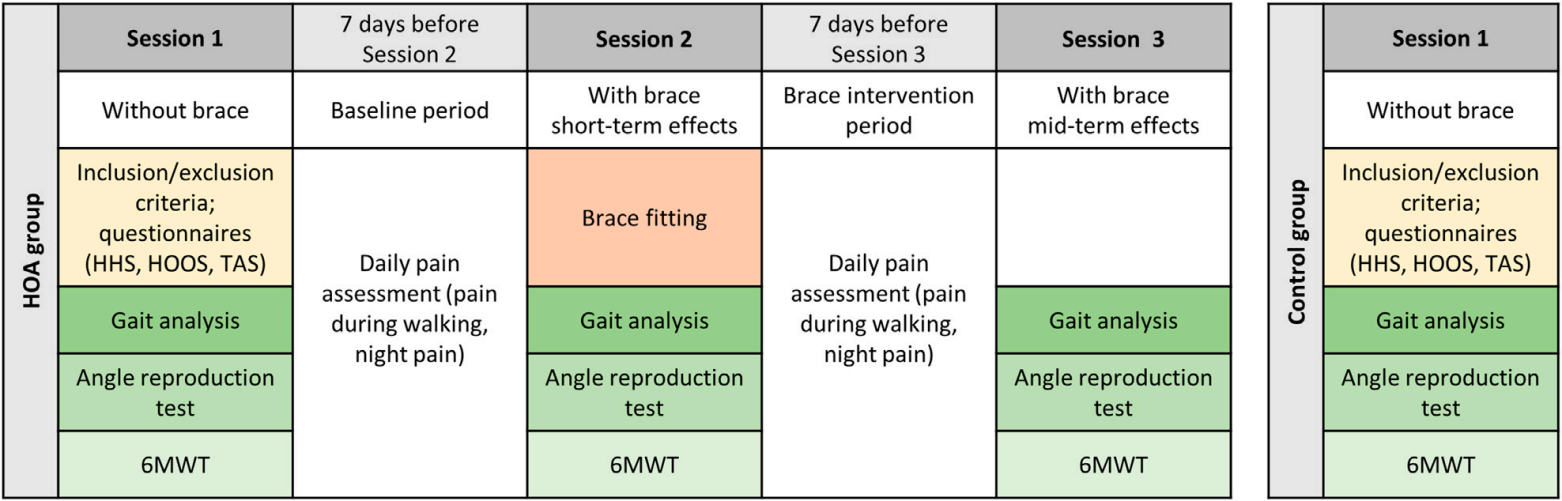
Study procedure for the HOA and control groups. HHS = Harris Hip Score, HOOS = Hip Osteoarthritis Outcome Score, TAS = Tegner Activity Score, 6MWT = 6-minute walking test.

Subjects of the HOA group were tested on three occasions. The time interval between sessions 1 and 2 was 5 ± 4 weeks, and between sessions 2 and 3 was 1 ± 0 weeks. Subjects of the control group were tested on one occasion, which was identical to the procedure during the first session of the HOA group (Figure 1).

Session 1: During the first session, subjects were screened for the inclusion and exclusion criteria and completed two questionnaires concerning hip function (Harris Hip Score (Harris, 1969) and Hip Osteoarthritis Outcome Score (Klässbo et al., 2003)) and one questionnaire regarding their activity level (Tegner Activity Score (Tegner and Lysholm, 1985; Swanenburg et al., 2014)). Anthropometrical measures for biomechanical modelling as well as fitting of the brace were taken. Afterwards, subjects were equipped with retroreflective markers. After a standardized instruction and a familiarization trial, subjects completed the proprioception test followed by the biomechanical gait analysis. Thereby, subjects walked barefoot at their self-selected speed for five valid trials (Laroche et al., 2011). For a valid trial, subjects had to strike the first force plate entirely with the ipsilateral foot and the second force plate entirely with the contralateral foot. A constant walking speed was controlled using light barriers allowing a difference of ±5% from the first valid trial. Finally, subjects completed the 6MWT.

Baseline Period: Subjects recorded their pain perception during walking and at night using a standardized protocol with VAS scales on a daily basis for 7 days.

**FIGURE 2:**
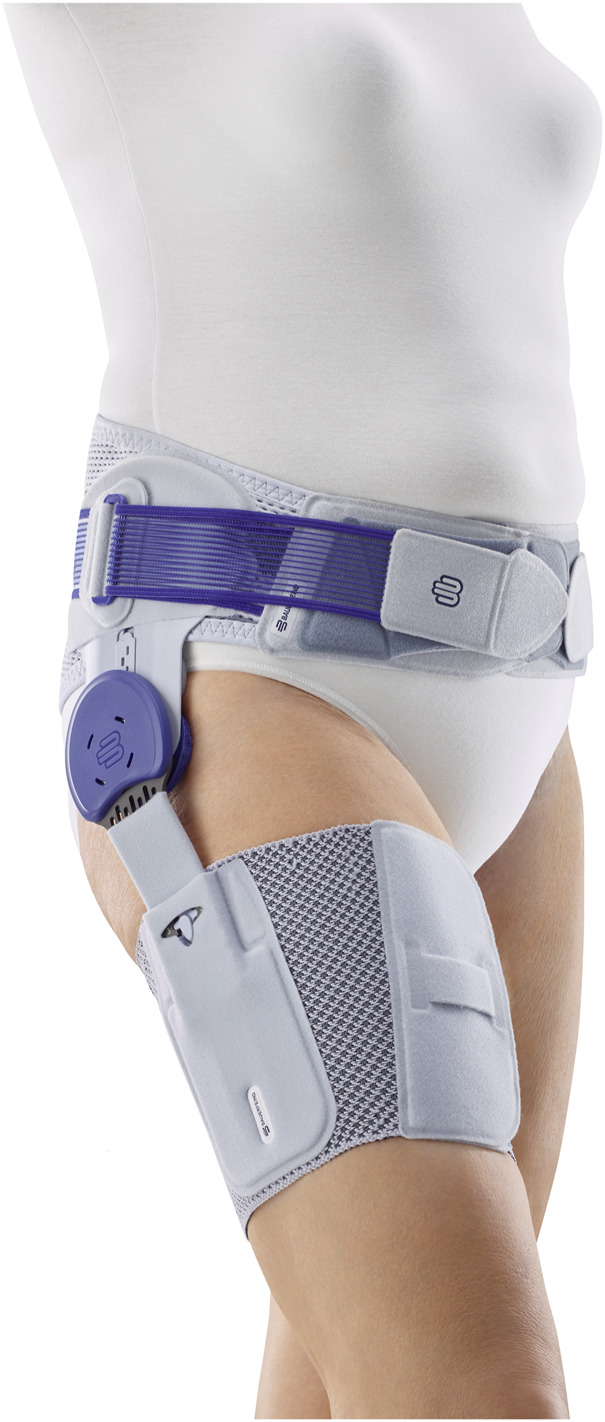
CoxaTrain hip brace, ©Bauerfeind AG.

Session 2: In the second session, subjects were individually fitted with a hip brace (CoxaTrain, Bauerfeind AG, Zeulenroda-Tribes, Germany) by an experienced orthopaedic technician. The hip brace consists of a pelvis belt equipped with a gluteal pad and two friction pads at the iliosacral joints, an aluminium joint splint including a moving trochanter pad and a thigh bandage (Figure 2). The brace aims at stabilizing the pelvis through the tight fitting pelvis belt and to stimulate several trigger points at the gluteus and iliosacral joints. Additionally, the moving trochanter pad applies a friction massage to the muscle insertions at the greater trochanter to relax hypertonic muscles and thereby increase hip joint mobility.

After a short period of brace familiarization (autonomous walking with the brace throughout the lab until the subjects felt comfortable) the procedure of session 1 was replicated. As the pelvis belt of the brace covers the anatomical landmarks for marker placement at the pelvis, retroreflective markers had to be placed on the brace (left and right anterior superior iliac spine) or were attached through the mesh fabric (left and right posterior superior iliac spine).

Brace Intervention Period: Subjects were instructed to wear the brace during their daily activities for 1 week for at least 4 h per day. Subjects recorded the brace wear time on a standardized form and, additionally, the brace was equipped with a thermal sensor (*Orthotimer®,* Rollerwerk Medical Engineering and Consulting, Balingen, Germany) to objectively detect brace wear time. Again, subjects recorded their pain perception during walking and at night using a standardized protocol on a daily basis.

Session 3: The third test session was conducted in the same way as in session 2.

### 2.4 Data Processing and Biomechanical Modelling

Kinematic and GRF data of the walking trials were filtered using a 2^nd^ order Butterworth low pass filter with a cut-off frequency of 15 Hz (Kristianslund et al., 2012). Kinematic data of the proprioception trials were filtered using a 2^nd^ order Butterworth low pass filter with a cut-off frequency of 6 Hz (Roithner et al., 2000).

An inverse kinematics and dynamics approach using the multi-body model ALASKA Dynamicus (Härtel and Hermsdorf, 2006) was used to calculate 3D joint angles and external joint moments. The location of the hip joint centre was calculated based on the formula proposed by Harrington et al. (2007) and Kainz et al. (2015). Hip joint angles were defined as the rotation of the femur relative to the pelvis coordinate system (three Bryant angles). The axes of the pelvis coordinate system are defined by the two anterior superior iliac spine (ASIS) markers (1), the midpoints between the two ASIS and two posterior superior iliac spine (PSIS) markers and orthogonal to the plane through ASIS and PSIS markers (3).

Data post-processing was conducted using Matlab (R2017b). Joint angles were time normalized to one gait cycle (100 time points) beginning with the heel strike on the force plate until the next consecutive heel strike of the same foot. Heel strike on the force plate was determined when the vertical GRF exceeded a threshold of 10 N (Tirosh and Sparrow, 2003). The consecutive heel strike was detected using the vertical trajectory of the heel marker. Joint moments were normalized to bodyweight as well as time normalized to the stance phase (100 time points). Normalized angle and moment curves were averaged across the five trials of each condition. Lastly, peak hip joint angles, hip joint ROM as well as peak hip joint moments were calculated and used as dependent variables.

Additionally, temporal-spatial gait parameters were analysed as dependent variables. Walking speed was calculated as the mean velocity of the centre of mass (COM) in the anterior-posterior direction across each stride. Stride and step length were calculated as the distance between the heel marker position at the first and second heel strike of the ipsilateral foot or at the first heel strike of the ipsilateral and contralateral foot, respectively, in the walking direction.

As a parameter for hip proprioception the absolute angle error (AAE) was defined as the absolute difference between the hip flexion angles in the initially adopted and the resumed position (Arvin et al., 2015) (mean across 0.1 s from instant of switch pressing). Thus, lower error values represent a better hip proprioception. AAEs were calculated for each subject and averaged across the five trials.

Pain perception during walking as well as night pain were obtained daily via questionnaires using VAS in the baseline and intervention periods and averaged across the 7 days.

### 2.5 Statistics

All statistical tests were performed using IBM SPSS Statistics 25.0 (IBM Corporation, Armonk, NY, United States). All variables were tested for normal distribution using the Shapiro-Wilk-Test. If normal distribution could not be assumed, nonparametric statistical tests were applied.

Firstly, differences between the HOA group at baseline and control group (1 time point, 2 groups, 1 degree of freedom) were analysed using t-tests for independent samples (or Mann-Whitney-U tests (MWU)).

Secondly, comparisons within the HOA group und different bracing conditions (3 time points, 2 degrees of freedom) were conducted using univariate ANOVAs for repeated measures (or Friedman tests). If sphericity was violated, Greenhouse-Geisser estimates were used to correct for these violations. For significant results in the ANOVA, a post-hoc analysis was conducted using t-tests for dependent samples (or Wilcoxon tests) with Holm-Bonferroni corrections to adjust for multiple comparisons.

For all statistical tests, the level of significance was set *a priori* to 0.05. To estimate the effect sizes for between-group comparisons, Cohen’s d was calculated based on means and standard deviations. Effect sizes were interpreted as |d| > 0.2 being a small effect, |d| > 0.5 a moderate effect and |d| > 0.8 a large effect (Cohen, 1988). Effect sizes for comparisons within the HOA group were calculated using partial eta squared (small effect: ɳ_p_^2^ ≥ 0.01; medium effect: ɳ_p_^2^ ≥ 0.06; large effect: ɳ_p_^2^ ≥ 0.14) (Cohen, 1988; Richardson, 2011). For analyses conducted with the Friedman test no effect sizes are presented.

Due to a technical error the force plate only recorded GRF in the vertical direction for two subjects in the control group. Therefore, these datasets were excluded during the analysis of joint moments. As the data can be classified as missing completely at random (Rubin, 1976) no corrections were made. Additionally, due to a technical issue the thermal sensors did not record the brace wear time for two subjects. Calculation of average wear times was based on the questionnaire data for those two subjects.

## 3 Results

There were no significant differences in the subject characteristics between the HOA and control groups concerning age, height, body mass, BMI or activity level (see Table 2).

### 3.1 Effects of Hip Osteoarthritis on Functional Capacity, Hip Proprioception and Gait Biomechanics

The HOA group covered a significantly lower distance during the 6MWT (559.7 ± 84.4 m) compared to the control group (631.9 ± 54.8 m) (*p* = 0.002; |d| = 1.01). No significant group effects on the AAE during the angle reproduction test were found (HOA: 3.93 ± 1.82°; CG: 3.80 ± 2.00°; *p* = 0.822; |d| = 0.07).

Subjects from the HOA group (1.23 ± 0.17 m/s) walked significantly more slowly than subjects from the control group (1.32 ± 0.11 m/s) (*p* = 0.049; |d| = 0.63) and showed a significant reduction in step length (HOA: 0.62 ± 0.07 m; CG: 0.67 ± 0.06 m; *p* = 0.013; |d| = 0.81). There were no significant differences between groups regarding absolute (HOA: 0.64 ± 0.05 s; CG: 0.64 ± 0.05 s; *p* = 0.774; |d| = 0.09) and relative (HOA: 62.42 ± 1.93%; CG: 62.9 ± 2.46%; *p* = 0.89; |d| = 0.22) stance phase duration, absolute (HOA: 0.39 ± 0.02 s; CG: 0.38 ± 0.04 s; *p* = 0.589; |d| = 0.28) and relative (HOA: 37.58 ± 1.93%; CG: 37.1 ± 2.46%; *p* = 0.89; |d| = 0.22) swing phase duration or stride length (HOA: 1.26 ± 0.13 m; CG: 1.33 ± 0.15 m; *p* = 0.061; |d| = 0.50).

Results for kinematics and dynamics during walking are presented in Table 3. The HOA group showed a reduced ROM in the sagittal and transverse planes. Hip joint kinematics and moments were reduced in the sagittal plane (peak extension, peak hip extension and flexion moment) for the HOA group. Additionally, the HOA group showed a reduced peak adduction and peak external rotation moment. Finally, an increase in pelvic tilt ROM was observed in the HOA group.

**TABLE 3:** Mean values and standard deviations (sd) of discrete hip and pelvis joint angles and hip joint moment gait parameters for the hip osteoarthritis (HOA) and control group (CG) with respective *p*-values and effect sizes (Cohen’s d) as revealed by independent sample t-tests/Mann-Whitney-U tests (MWU). Level of significance ≤0.05; * marks a significant result.

Movement plane	Variable	HOA without brace	CG without brace	*t*-test/MWU
		Mean (sd)	Mean (sd)	*p* (|d|)
Sagittal plane	Minimum angle/peak extension [°]	−22.55 (6.28)	−29.10 (4.54)	<0.001* (1.20)
	Maximum angle/peak flexion [°]	14.38 (5.84)	15.99 (5.54)	0.366 (0.28)
	Range of motion [°]	36.94 (8.30)	45.09 (5.43)	0.001* (1.16)
	Peak ext. hip extension moment [Nm/kg]	0.53 (0.19)	0.65 (0.16)	0.038* (0.68)
	Peak ext. hip flexion moment [Nm/kg]	−0.69 (0.18)	−0.83 (0.17)	0.008* (0.80)
	Pelvic tilt ROM [°]	4.97 (2.22)	3.58 (0.87)	0.012* (0.82)
Frontal plane	Minimum angle/peak adduction [°]	−7.65 (2.30)	−9.04 (2.40)	0.063 (0.59)
	Maximum angle/peak abduction [°]	4.83 (2.47)	4.54 (2.47)	0.710 (0.12)
	Range of motion [°]	12.48 (2.97)	13.58 (2.20)	0.180 (0.42)
	Peak ext. abduction moment [Nm/kg]	0.19 (0.08)	0.20 (0.08)	0.592 (0.13)
	Peak ext. adduction moment [Nm/kg]	−0.92 (0.10)	−1.04 (0.15)	0.010* (0.95)
	Pelvic obliquity ROM [°]	6.31 (2.16)	6.57 (2.08)	0.687 (0.12)
Transverse plane	Minimum angle/peak int. rotation [°]	−6.71 (10.84)	−11.48 (13.06)	0.428 (0.40)
	Maximum angle/peak ext. rotation [°]	5.47 (10.34)	4.19 (13.07)	0.726 (0.11)
	Range of motion [°]	12.18 (3.08)	15.67 (3.28)	0.001* (1.09)
	Peak ext. internal rotation moment [Nm/kg]	−0.07 (0.04)	−0.10 (0.10)	0.379 (0.40)
	Peak ext. external rotation moment [Nm/kg]	0.20 (0.05)	0.25 (0.08)	0.035* (0.76)
	Pelvic rotation ROM [°]	10.41 (4.35)	10.79 (3.21)	0.359 (0.10)

In summary, as hypothesized the HOA group showed 1) reduced walking speed and step length, 2) reduced sagittal hip ROM and peak extension angle as well as 4) reduced functional capacity. In contrast, no effects of HOA on 3) hip proprioception were found.

### 3.2 Effects of Hip Bracing on Pain, Functional Capacity, Hip Proprioception and Gait Biomechanics

#### 3.2.1 Effects of Hip Bracing on Pain, Functional Capacity and Hip Proprioception

During the intervention period, subjects wore the brace on average for 10.1 (± 3.5) hours per day. The average perceived pain during walking activities significantly decreased during the intervention period (18.4 ± 18.1 mm) compared to the baseline period (25.7 ± 15.3 mm) (*p* = 0.006). Additionally, night pain was significantly reduced during the intervention period (13.9 ± 15.9 mm) compared to the baseline period (17.0 ± 17.6 mm) (*p* = 0.042). Pain reduction was reported by 18 out of 21 subjects for walking activities and 14 out of 21 subjects for night pain. For the subjects with a positive brace effect on pain, mean reduction in VAS pain score was 10.5 ± 7.9 mm or 45.5 ± 28.3% for walking activities and 6.2 ± 5.4 mm or 41.6 ± 30.7% for night pain. For the subjects with a negative effect on pain, the mean increase was 11.4 ± 8.2 mm or 40.8 ± 37.5% for walking activities and 3.1 ± 3.2 mm or 84.2 ± 141.3% for night pain.

The distance covered during the 6MWT significantly increased after mid-term brace application (589.1 ± 82.7 m) compared to the baseline condition (559.7 ± 84.4 m) (*p* < 0.001) as well as compared to short-term brace application (562.3 ± 80.9 m) (*p* < 0.001). The level of hip pain before (*p* = 0.049, post-hoc analysis not significant) and after (*p* = 0.363) the 6MWT was not influenced by the bracing condition. No significant bracing effects on the AAE during the angle reproduction test were found (HOA without brace: 3.93 ± 1.82; HOA short-term: 3.37 ± 1.70; HOA mid-term: 3.57 ± 1.69; *p* = 0.397).

In summary, as hypothesized brace application resulted in 5) reduced pain perception as well as 7) increased functional capacity. As expected, the 11) increase in functional capacity occurred only after mid-term brace application. In contrast, no brace effects on 5) pain during the 6MWT or on hip proprioception were found, neither 10) after short-term nor after mid-term brace application.

#### 3.2.2 Effects of Hip Bracing on Gait Biomechanics

Effects of brace application on temporal-spatial gait parameters and discrete hip angle and hip moment parameters are displayed in Tables 4, 5, respectively. Joint angle and joint moment time curves are shown in the Supplementary Material. Mid-term brace application resulted in a significant increase in gait velocity, stride and step length compared to the unbraced and short-term conditions. Additionally, stance phase duration decreased after mid-term brace application compared to the unbraced condition.

**TABLE 4:** Mean values and standard deviations (sd) of temporal-spatial gait parameters for the HOA group with respective p-values and effect sizes as revealed by one-way repeated measures ANOVAs/Friedman tests and Holm-Bonferroni corrected pairwise comparisons. Level of significance ≤ 0.05; * marks a significant result; † marks analysis with a Friedman test.

Variable	Without brace	Short-term	Mid-term	ANOVA/Friedman	Without vs. short-term	Without vs. mid-term	Short-term vs. mid-term
	Mean (sd)	Mean (sd)	Mean (sd)	*p* ( $\eta_{p}^{2}$ )	*p*	*p*	*p*
Gait velocity [m/s]	1.23 (0.17)	1.26 (0.21)	1.31 (0.21)	0.001* (0.29)	0.153	0.003*	0.032*
Stance phase duration [s]	0.64 (0.05)	0.64 (0.05)	0.62 (0.06)	0.004*†	0.244	0.006*	0.06
Swing phase duration [s]	0.39 (0.02)	0.38 (0.02)	0.38 (0.02)	0.469 (0.03)			
Stance phase duration [%]	62.42 (1.93)	62.38 (1.78)	61.92 (1.88)	0.151 (0.10)			
Swing phase duration [%]	37.58 (1.93)	37.62 (1.78)	38.08 (1.88)	0.151 (0.10)			
Stride length [m]	1.26 (0.13)	1.27 (0.15)	1.30 (0.14)	0.003* (0.26)	0.271	0.003*	0.032*
Step length [m]	0.62 (0.07)	0.63 (0.08)	0.64 (0.07)	0.006* (0.23)	0.334	0.009*	0.026*

**TABLE 5:** Mean values and standard deviations (sd) of discrete joint angle and joint moment gait parameters for the HOA group with respective p-values and effect sizes as revealed by one-way repeated measures ANOVAs/Friedman tests and Holm-Bonferroni corrected pairwise comparisons. Level of significance ≤ 0.05; * marks a significant result; † marks analysis with a Friedman test.

Movement plane	Variable	Without brace	Short-term	Mid-term	ANOVA/Friedman	Without vs. short-term	Without vs. mid-term	Short-term vs. mid-term
		Mean (sd)	Mean (sd)	Mean (sd)	*p* ( $\eta_{p}^{2}$ )	*p*	*p*	*p*
Sagittal plane	Minimum angle/peak extension[°]	−22.55 (6.28)	−23.69 (6.05)	−23.71 (5.80)	0.405†			
	Maximum angle/peak flexion [°]	14.38 (5.84)	11.77(5.47)	11.97 (7.14)	0.011* (0.20)	0.006*	0.052	0.85
	Range of motion [°]	36.94 (8.30)	35.47 (8.34)	35.67 (8.76)	0.039* (0.17)	0.078	0.140	0.594
	Peak ext. hip extension moment [Nm/kg]	0.53 (0.19)	0.61 (0.21)	0.65 (0.21)	0.005* (0.26)	0.006*	0.006*	0.299
	Peak ext. hip flexion moment [Nm/kg]	−0.69 (0.18)	−0.64 (0.21)	−0.65 (0.25)	0.469 (0.04)			
	Pelvic tilt ROM [°]	4.97 (2.22)	6.15 (2.53)	6.00 (2.47)	<0.001* (0.32)	<0.001*	0.012*	0.634
Frontal plane	Minimum angle/peak adduction [°]	−7.65 (2.30)	−7.18 (2.38)	−7.37 (2.76)	0.432 (0.04)			
	Maximum angle/peak abduction [°]	4.83 (2.47)	4.62 (2.48)	4.42 (2.59)	0.516 (0.03)			
	Range of motion [°]	12.48 (2.97)	11.80 (2.68)	11.79 (3.17)	0.096 (0.11)			
	Peak ext. abduction moment [Nm/kg]	0.19 (0.08)	0.19 (0.10)	0.21 (0.09)	0.055†			
	Peak ext. adduction moment [Nm/kg]	−0.92 (0.10)	−0.95 (0.14)	−0.94 (0.17)	0.614 (0.02)			
	Pelvic obliquity ROM [°]	6.31 (2.16)	6.68 (2.02)	6.59 (1.91)	0.329 (0.05)			
Transverse plane	Minimum angle/peak int. rotation [°]	−6.71 (10.84)	−6.35 (10.25)	−4.50 (10.27)	0.172†			
	Maximum angle/peak ext. rotation [°]	5.47 (10.34)	5.88 (9.62)	9.37 (10.76)	0.077 (0.12)			
	Range of motion [°]	12.18 (3.08)	12.23 (3.25)	13.87 (4.49)	0.023* (0.17)	0.937	0.069	0.069
	Peak ext. internal rotation moment [Nm/kg]	−0.07 (0.04)	−0.07 (0.03)	−0.07 (0.03)	0.565†			
	Peak ext. external rotation moment [Nm/kg]	0.20 (0.05)	0.21 (0.06)	0.22 (0.07)	0.280 (0.06)			
	Pelvic rotation ROM [°]	10.41 (4.35)	11.37 (3.95)	12.49 (5.41)	0.001*†	0.026*	0.001*	0.026*

In the sagittal plane, brace application reduced the peak flexion angle after short-term application and increased the peak extension moment in both braced conditions. Additionally, pelvic tilt and pelvic rotation ROM increased with brace application.

In summary, as hypothesized brace application resulted in 9) an increase of step length and walking speed. As expected 11), both changes occurred only after mid-term brace application. However 8), no brace effects on hip sagittal ROM or peak extension angle were found, neither 11) after short-term nor after mid-term brace application.

## 4 Discussion

The present study first aimed to quantify the effects of moderate unilateral HOA on gait biomechanics, proprioception and functional capacity. The main findings are that, in line with our hypotheses, subjects with HOA walked significantly more slowly with a reduced step length, sagittal hip ROM and peak extension angle and had a reduced functional capacity. In contrast to our expectations, no effect of HOA on hip proprioception was found.

The second aim of this study was to evaluate the short- and mid-term effects of a hip brace on gait biomechanics, hip proprioception, pain perception and functional capacity. In summary, as hypothesized, after mid-term brace application step length, walking speed and functional capacity were significantly increased. Additionally, pain perception was significantly lower in the intervention period. However, no brace effects on hip sagittal ROM, peak extension angle or hip proprioception were found, neither after short-term nor after mid-term brace application.

### 4.1 Effects of Hip Osteoarthritis on Functional Capacity, Hip Proprioception and Gait Biomechanics

The effects of HOA on gait biomechanics observed in this study were alterations of the gait pattern that have been previously described (e.g. Watelain et al., 2001; Constantinou et al., 2017). Thus, the present HOA group presented typical symptoms of HOA gait. Walking speed in the HOA subjects was significantly lower than the CG with a mean walking speed of 1.23 m/s, representing a difference of 6.8%. This is a smaller difference than reported by Constantinou et al. (2014) who, in a meta-analysis, calculated a mean walking speed of 0.95 m/s and a mean difference of 26% compared to the CG. However, subjects with end-stage HOA scheduled to undergo a total hip replacement were included in their analysis. The authors attributed the reduction in gait speed to a reduction in step length of the affected limb (Constantinou et al., 2014), which was also observed in the present study. Additionally, our subjects showed limited mobility in hip extension and hip transverse rotation movement. The limitations in dynamic ROM have previously been attributed either to limitations in passive joint mobility (Holla et al., 2011; Eitzen et al., 2012; Baker et al., 2016) or as a strategy to reduce joint loading and pain (Hurwitz et al., 1997; Ornetti et al., 2011; Meyer et al., 2018).

For both subject groups peak sagittal hip angles observed in this study deviated from those reported previously by e.g. Eitzen et al. (2012) Ornetti et al. (2011) or Tateuchi et al. (2014). While the ROM of 45° (control group) is comparable to the data presented by these authors the peak values are shifted towards lower hip flexion and more pronounced hip extension. This offset in sagittal hip rotation was previously described by authors comparing different biomechanical models and is likely to be caused by differences in the definition of pelvis neutral position (Roelker et al., 2017; Falisse et al., 2018). Consequently, deviations in hip joint angles must be interpreted with caution when comparing different studies and individual model definitions have to be considered in this context.

The limitations observed for hip joint excursion were reflected in reduced external hip flexion, extension, adduction and external rotation moments. This is in line with data from Tateuchi et al. (2021) who reported a reduction of all peak joint moments with decreased step length. The reduction in peak adduction moment has also been reported in a recent meta-analysis of HOA walking dynamics (Diamond et al., 2018). However, the authors suggested that the alterations only occur in subjects with end-stage HOA. One reason for this might be that the studies included in the meta-analysis used matched walking speeds or statistical methods to correct for different walking speeds between the healthy and the HOA groups. As hip adduction moment correlates with walking speed (Rutherford and Hubley-Kozey, 2009) this might artificially lower the hip adduction moment observed for healthy control subjects. However, during natural everyday walking conditions, joint moments are lower even in subjects with mild to moderate HOA as seen in this study. Thereby, the peak adduction moment is thought to be a key parameter for hip joint loading (Wesseling et al., 2015) as it has to be counteracted by the hip abductor muscles. Tateuchi et al. (2017) reported that the daily cumulative hip moment in the frontal plane in particular and potentially the cumulative hip moment in the sagittal plane are predictors of radiographic progression of HOA. Thus, reduced hip joint moments are likely to origin in pain avoidance gait strategies adopted by the subjects and decreased muscular function (Watelain et al., 2001; Ornetti et al., 2011; Meyer et al., 2018).

Beside the changes in hip joint biomechanics, the ROM of the pelvis in the sagittal plane (pelvis tilt) was significantly increased. Similar results were found by Watelain et al. (2001) in a group of people with early-stage HOA. Lee et al. (1997) stated that “anterior pelvic tilting was the most closely associated compensatory mechanism for reduced hip extension during gait.” In contrast, pelvis motion was not a predominant gait feature of HOA gait in a study by Meyer et al. (2015).

The decreased walking speed and step length resulted in a significant reduction of functional capacity assessed using the 6MWT. Despite a comparable HHS, the distances covered during this study by either the HOA or the control group were lower than those reported by Eitzen et al. (2015) and Rydevik et al. (2010) who reported distances between 630 and 673 m for HOA subjects and 719 m for healthy control subjects. One explanation for this might be different test settings with a circuit of 54 m length in our study and a corridor of 20 m length in the studies of Eitzen et al. (2015) and Rydevik et al. (2010). Likewise, Kumar et al. (2015) reported a value of 628 m for patients with mild to moderate HOA. They, however, defined HOA solely based on radiographic imaging and did not include functional criteria; and the reported HOOS values for pain and activities of daily living were higher than in the present study representing less pain and functional impairments.

To our knowledge, this was the first study quantifying hip proprioception in a cohort of people with HOA. The applied angle reproduction test did not reveal any significant effects of HOA on sagittal plane hip proprioception. This result might either indicate that, in contrast to KOA, HOA does not cause a loss in proprioceptive perception, or that the measurement technique applied in this study was unable to detect any differences. Pickard et al. (2003) compared the hip joint position sense of younger and older adults using an angle reproduction test in the frontal plane and likewise did not find any significant differences between the groups. Thus, in contrast to knee joint position sense, for which a decline with age has frequently been reported (Kaplan et al., 1985; Hurley et al., 1998), no age effect was detectable for the hip joint. Hillier et al. (2015) stated that active angle reproduction tests, while reflecting the functional use of proprioception, requires sufficient kinaesthetic memory of the pre-established position as well as motor control to readopt the joint position. As hip flexion movement was performed in a one-legged standing position, maintaining balance as well as flexing the hip joint against gravity might have required a large portion of motor control. Therefore, functional limitations in this area might have masked underlying differences in the sensory quality of hip proprioception. Performing the hip flexion angle reproduction test supine might eliminate this interference.

### 4.2 Effects of Hip Bracing on Pain, Functional Capacity, Hip Proprioception and Gait Biomechanics

The application of a hip brace had two aims: 1) stabilizing the pelvis and stimulating several trigger points at the gluteus and iliosacral joints and 2) mobilizing the hip joint by applying a friction massage to the muscle insertions at the greater trochanter. Application resulted in a significant reduction of the perceived pain during walking activities and at night. Thereby, 18 out of 21 subjects reported a pain reduction during walking activities and 14 out of 21 subjects reported a decrease in night pain. In contrast, brace application in the study of Nérot and Nicholls (2017) only resulted in pain reduction in 9 of 14 subjects. They, however, only analysed immediate pain relief. In a longitudinal study on the effects of the WISH type of S-form hip brace, pain perception decreased the most in the first 3 months of brace use (Sato et al., 2008). Thus, longer brace application might further increase the positive effects.

The observed absolute changes in VAS during this study of 10.5 mm (subjects with less pain) and 11.4 mm (subjects with more pain) for walking activities as well as 6.2 mm (subjects with less pain) and 3.1 mm (subjects with more pain) for night pain are rather small. A study intending to find the minimal clinically important difference (MCID) in a cohort of subjects with HOA reports values of 15.3 mm or 32% (Tubach et al., 2005). Thus, in absolute values the clinical relevance of the shown improvements or deteriorations is questionable. However, the relative changes (less pain: 45.5% and more pain: 40.8% for walking activities; less pain: 41.6% and more pain: 84.2% for night pain) exceed the proposed MCID of 32%. Hence, brace-induced changes in pain perception might still be perceived as beneficial or detrimental. Additionally, Dekker (2019) states that anchor-based methods to detect MCIDs yield highly variable results. The author further stresses that the MCID is highly subjective and that the individual patient has to decide which improvement is important enough to undergo a treatment. Further analyses of gait biomechanics at an individual level might provide additional insights on why, in a small portion of subjects, brace application leads to an increase in pain.

Besides the changes in pain perception, subjects were able to increase their step and stride length as well as walking speed which resulted in an increased performance during the 6MWT. The distance covered during the 6 min increased on average by 5%. In a study with people with KOA it was shown that performance in the 6MWT was highly correlated with the KOA outcome score subscales for pain and quality of life (Ateef et al., 2016). Despite the increase in walking distance covered during the 6MWT, the pain level after the task remained unchanged. Thus, functional capacity increased without negative effects on pain perception. Sato et al. (2012) analysed hip brace effects using the timed up and go test and, similarly, found increases in functional capacity. Furthermore, the improvement increased after long-term brace use. Thus long-term brace usage might further increase the positive effects on functional capacity observed after mid-term brace application.

In contrast to our expectations, no effect of bracing on hip proprioception was observed. While this contradicts some findings published for knee bandages (Beynnon et al., 1999, 2002; Selfe et al., 2008, 2011; Baltaci et al., 2011; Bodendorfer et al., 2019), it is not surprising with regard to the fact that no reduction in joint position sense was found for the HOA group. However, positive brace effects have also been found in healthy young people who should not show proprioceptive loss (Baltaci et al., 2011). Therefore, again, the testing procedure might not be sensitive enough to detect smaller changes in hip joint position sense.

Brace application overall did not lead to a “normalization” of the gait patterns in that typical symptoms of HOA (e.g. decreased hip extension or reduced sagittal ROM) remain even after mid-term brace application. This is in line with results from patients treated with total hip replacement (THR). Beaulieu et al. (2010) showed that alterations in gait biomechanics such as reduced peak flexion angle, peak extension angle and sagittal plane ROM remained even 10 months after surgery. Similar results were found by Foucher et al. (2007) as well as Zügner et al. (2018) even one and 2 years after THR, respectively. Beaulieu et al. (2010) and Foucher et al. (2007) speculated that pain-avoidance strategies adopted pre-operatively might persist even after successful surgery, or that alterations are caused by persistent muscle weaknesses. The intervention period of 1 week might therefore not have been enough time for the subjects to adopt a normal gait pattern.

Brace application, in contrast, induced some additional biomechanical changes. Short-term brace application resulted in a significant reduction of the peak hip flexion angle, and there is a tendency of this effect to remain even after mid-term brace application. This reduction of the peak flexion angle might reflect a passive resistance of the brace to the hip flexion movement. Nérot and Nicholls (2017) reported that brace application led to a feeling of either restriction or support by the subjects. Unfortunately, no kinematic data on hip flexion was presented.

Brace application not only influenced hip but also pelvis kinematics. In both braced conditions pelvic tilt and rotation ROM were increased in comparison with the unbraced condition. Anterior tilting of the pelvis as well as more pronounced rotation of the pelvis might allow the patients to increase step length despite limitations in hip extension and internal rotation mobility (Leigh et al., 2016). Liang et al. (2014) demonstrated that for gait velocities above 1.0 m/s pelvic rotation lengthens the step which is accompanied by an increase in pelvic rotation ROM.

As the pelvis is closely connected to the lumbar spine (Thurston and Harris, 1983; Whittle and Levine, 1999; Ike et al., 2018) alterations in pelvis motion are likely to affect lumbar spine mobility (Hurwitz et al., 1997; Watelain et al., 2001). People with HOA frequently report lower back pain (Thurston, 1985). Previous studies on lower back pain showed an association between pain and increased pelvic horizontal rotation (Huang et al., 2011). In contrast, people with acute lower back pain show reduced pelvic rotation, perhaps to reduce forces transmitted across the lumbar spine (Taylor et al., 2004). Thus, increased pelvic rotation without according lumbar spine movement might increase stress on the lumbar spine and the iliosacral joints (Watelain et al., 2001). However, increased pelvic and lumbar spine motion is not correlated with back pain in patients with HOA (Thurston, 1985). The applied hip brace exerts pressure on the pelvis and onto several trigger points at the iliosacral joints. Future research should clarify whether increases in pelvic motion seen with brace application are enabled by a stabilization of the pelvis-spine complex as well as pain reduction; and whether increased pelvic motion causes greater stress of the lumbar spine, potentially triggering lower back pain with long-term brace usage.

The observed kinematic alterations were reflected in an increase of the external peak extension moment for both braced conditions in comparison to the unbraced condition. Tateuchi et al. (2021) reported an increase for all peak joint moments when gait velocity increased, especially when velocity increase was achieved by an increase in step length rather than cadence. However, despite an increase in gait velocity and step length, none of the other hip joint moments increased in this study. The peak of the extension moment is reached during terminal stance. A longer step length caused by a more forward tilted pelvis might increase the distance between the foot (and hence the centre of pressure) and the COM (Leigh et al., 2016). Thus, the GRF lever arm might be increased resulting in larger extension moments. Similarly, in a longitudinal study investigating the effects of a medical foot device in a population of people with HOA over 1 year, an increase in hip extension moment accompanied by a decrease in hip pain was found (Solomonow-Avnon et al., 2017). This is in line with results from Hurwitz et al. (1997) who reported a negative correlation of hip pain and peak hip extension moment. Thus, the pain relief caused by brace application might have enabled the HOA patients to increase the hip extension moment. Furthermore, application of the WISH-type hip brace resulted in larger vertical GRF during walking (Yamaji et al., 2009). Although no joint moments were reported they are likely to increase with increasing GRF (Toda et al., 2015).

In summary, this study demonstrated a positive effect of hip brace application on hip pain and functional capacity in a cohort of people with mild-to-moderate unilateral HOA. Thereby, patients in our study demonstrated typical biomechanical features of HOA gait. Brace application increased walking speed, step length and external peak extension moment. Additionally, brace application impacted pelvis mobility with increased ROMs in the sagittal and transverse planes. As the applied brace does not intend to mechanically alter hip joint biomechanics but rather influence soft tissue structures surrounding the hip joint, not only immediate but also mid-term effects were seen. Lastly, it was found that neither HOA nor hip bracing altered hip proprioception in the sagittal plane.

### 4.3 Limitations

Alongside the strengths of our study, there are also some limitations.

1) The order of the bracing conditions in the HOA group was not randomized, raising the risk for potential sequencing effects. Mild to moderate HOA is characterized by alternating phases of less and more pain (Bastick et al., 2016). Thus, to minimize the effects of pain fluctuation on the study results, we intended to have brief intervals between the test sessions. As the main focus of the hip brace is manipulation of soft tissue, application might cause long-term effects beyond the period of application, and an according wash-out period between test sessions would have been necessary. Therefore, randomization of bracing conditions was not conducted. Additionally, it has to be noted that due to the duration of brace production and availability of the subjects the time interval between session 1 and session 2 was substantially longer (5 ± 4 weeks) than the time interval between session 2 and session 3 (1 ± 0 weeks).
2) Equally, for obvious reasons it was not possible to blind the subjects regarding the brace intervention which enables a possible placebo effect, especially on subjective parameters such as pain perception.
3) For the biomechanical gait analysis, subjects were equipped with retroreflective markers on the pelvis. In sessions 2 and 3 subjects wore the brace during the measurements and, thus, the anterior and posterior iliac spines were covered by the pelvis belt of the brace. Hence, markers of the anterior spine had to be placed on the hip belt while markers for the posterior spine were attached through the mesh fabric of the pelvis belt. Application of markers on clothing increases the risk of relative movement between the bony segment and the marker (Milner, 2008). The application of other methods such as clusters, often reported for knee brace analyses (Focke et al., 2020; Turner et al., 2021), is not applicable for the hip joint as the pelvis was entirely covered by the brace and adjoining segments (thighs and torso) possess large portions of wobbling mass. However, as the pelvis belt of the brace was fitted very tightly, relative movement between the pelvis and the brace is probably small but cannot be fully excluded.
4) The results of our study confirmed the often-reported effect of reduced gait velocity in patients with HOA. Additionally, gait velocity increased after mid-term brace application. Thus, gait velocity differed between the CG and the HOA group as well as within the HOA group under different bracing conditions. It has previously been shown that gait velocity correlates with hip joint kinematics and dynamics (Lelas et al., 2003; Fukuchi et al., 2019). Therefore, observed changes in gait biomechanics might reflect differences in gait velocity to some degree. Several methods, including prescribed walking speeds or statistical methods (e.g. the analysis of covariance (ANCOVA)), have been used to account for such different walking speeds (Astephen Wilson, 2012). However, the use of prescribed gait speeds contains the risk of not capturing normal gait patterns as people are forced to walk with prescribed speed and thus does not reflect e.g. joint loading on a daily basis (Astephen Wilson, 2012). Additionally, it has been shown that gait modifications in people with HOA persist even at matched gait speeds (Ismailidis et al., 2021). The application of ANCOVA neglects the fact that differences in gait speeds between groups are not a source of random error variability but rather representative of the population characteristics (Astephen Wilson, 2012). Therefore, no prescriptions or corrections regarding gait velocity were applied in this study as previously applied by others (Eitzen et al., 2012; Meyer et al., 2018).

### 4.4 Outlook

This was one of the first studies to present comprehensive data regarding the effects of hip bracing for patients with HOA on hip and pelvis biomechanics, proprioception, functional capacity and pain perception. Thereby, not only immediate but also mid-term effects were presented. Brace application resulted in several alterations of the gait biomechanics, functional capacity and pain perception. However, HOA as well as hip braces have been shown to impact the movement and loading at other body locations such as the knee (Hurwitz et al., 1997; Tateuchi et al., 2014; Rutherford et al., 2015; Nérot and Nicholls, 2017), ankle (Kubota et al., 2007; Ornetti et al., 2011; Schmitt et al., 2015) and lumbar spine (Murray et al., 1971; Thurston, 1985). Alterations occurring at the hip joint might therefore impact the function of other body locations and might trigger or prevent concomitant diseases such as KOA (Shakoor et al., 2014; Rutherford et al., 2015; van Drongelen et al., 2020). Therefore, future research should include analyses of the contralateral limb, adjacent joints or even whole body movement. Thereby the application of pattern recognition methods such as a principal component analysis or cluster analysis on whole body kinematics as proposed by Meyer et al. (2015), Stetter et al. (2020) and van Drongelen et al. (2021) might allow us to gain additional insights into HOA and brace effects in other joints or the entire body.

While the biomechanical effects of hip brace application remain partly unexplained, positive effects on pain perception and functional capacity are encouraging enough to expand the research on hip bracing for patients with mild to moderate HOA. Thereby, special attention should be paid to the long-term effects of brace application. As the brace used in the present study is not designed to mechanically alter hip joint movement but instead focuses on soft tissue manipulation, longer periods of brace application might further increase the effects already observed after 1 week. Therefore, analyses of brace effects on muscle activity during gait should be included as people with HOA have been shown to have decreased muscle strength (Loureiro et al., 2013).

### 4.5 Summary

The present study is one of the first comprehensive studies to quantify the kinematic, dynamic, proprioceptive and functional effects of a hip brace in a clearly defined cohort of subjects with mild to moderate unilateral HOA. Additionally, insights on the effects of HOA on gait biomechanics, hip proprioception and functional capacity were gained or extended. While hip proprioception was not influenced either by HOA or by hip bracing, there were substantial impacts of both parameters on functional capacity and gait biomechanics. Brace application resulted in reduced pain perception and higher functional capacity. However, as key parameters of HOA gait such as a reduced peak extension angle remained unchanged, the underlying mechanisms remain partly unclear. Future studies should therefore include additional data regarding whole-body biomechanics or muscle activation and extend the brace application period to gain insights on long-term brace effects.

## Data Availability

The raw data supporting the conclusion of this article will be made available by the authors, without undue reservation.
