# Supplementary material for: Effects of Hip Bracing on Gait Biomechanics, Pain and Function in Subjects With Mild to Moderate Hip Osteoarthritis
Source: Front Bioeng Biotechnol. 2022 Jul 11;10:888775. doi: 10.3389/fbioe.2022.888775 (PMC9309805; doi:10.3389/fbioe.2022.888775)
Supplement: Supplementary file 1 [file DataSheet1.docx]

Supplementary Material


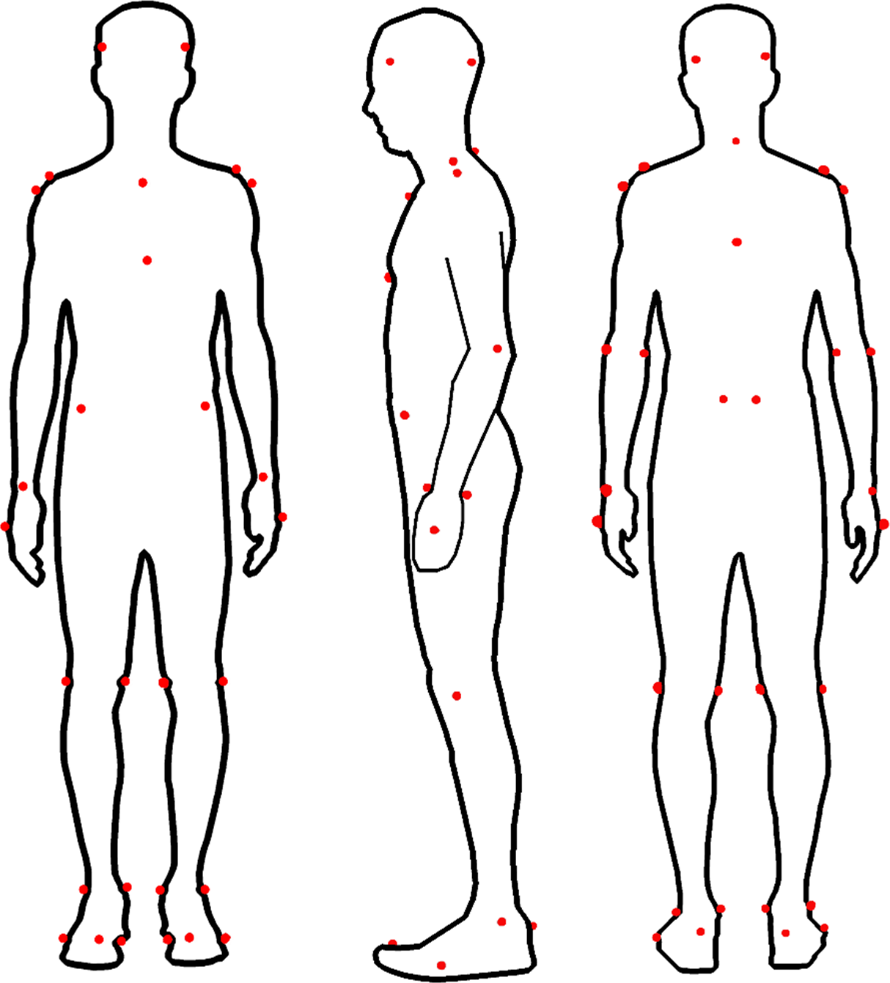


**Supplementary Figure 1.** Locations of the 42 retroreflective markers from frontal, side and rear view, respectively.

**
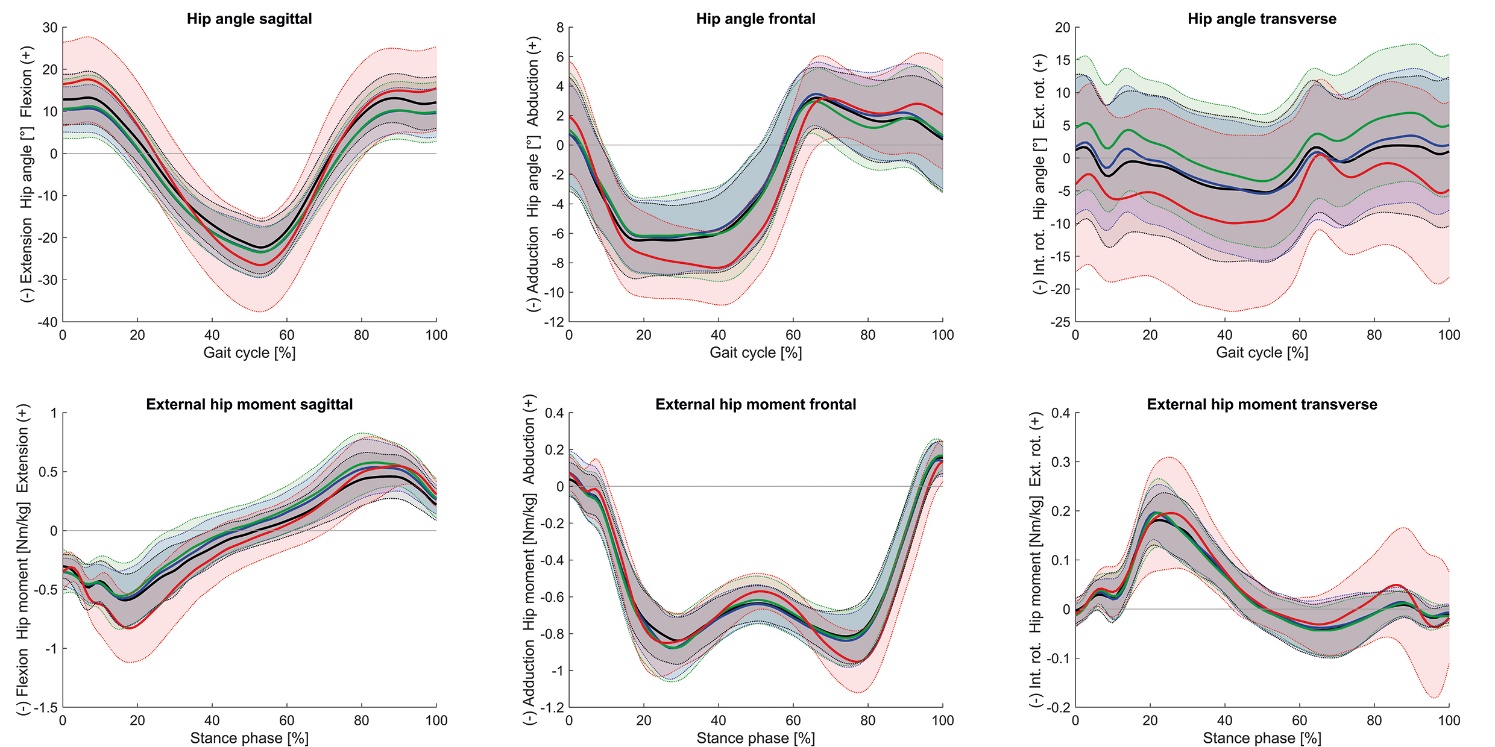
**

**Supplementary Figure 2.** Time curves (mean ± standard deviation) of hip joint angles [°] and moments [Nm/kg] normalized to gait cycle or stance phase. Red line = control group, black line = HOA group without brace, blue line = HOA group short-term, green line = HOA group mid-term.

**Supplementary Table 1.** Definitions of the locations of the 42 retroreflective markers.

| **Segment**  **(Number of markers)** | **Marker name** | **Description** |
| --- | --- | --- |
| Head (4) | LFHD / RFHD | Left & right forehead between os frontal and os parietal |
|  | LBHD / RBHD | In a horizontal line to the right / left forehead marker |
| Shoulder (6) | C7 | Spinous process of the 7^th^ cervical vertebrae |
|  | LACR / RACR | Left & right acromio-clavicular joint |
|  | LHUM / RHUM | Left & right humeral head |
|  | CLAV | Centred between the articuli sterno-clavicularis |
| Thorax (2) | STRN | Xiphoid process of the sternum |
|  | T10 | Spinous process of the 10^th^ thoracic vertebrae |
| Hip (4) | LASI / RASI | Left & right posterior superior iliac spine |
|  | LPSI / RPSI | Left & right posterior superior iliac spine |
| Upper arm (4) | LELBlat / RELBlat | Left & right lateral epicondyle of the humerus |
|  | LELBmed / RELBmed | Left & right medial epicondyle of the humerus |
| Forearm (4) | LWRIlat / RWRIlat | Left & right ulna-styloid process |
|  | LWRImed / RWRImed | Left & right radius-styloid process |
| Hand (2) | LFIN / RFIN | Dorsal side of the proximal third metacarpal bone |
| Leg (4) | LKNElat / RKNElat | Left & right epicondylus lateralis femoris |
|  | LKNEmed / RKNEmed | Left & right epicondylus medialis femoris |
| Ankle (6) | LMALlat / RMALlat | Fibula apex of the lateral malleolus |
|  | LMALmed / RMALmed | Tibia apex of the medial malleolus |
|  | LHEEL / RHEEL | Posterior surface of the calcaneus |
| Foot (6) | LFOOTlat / LFOOTmed | First metatarsophalangeal joint |
|  | LFOOTmed / RFOOTmed | Fifth metatarsophalangeal joint |
|  | LTOE / RTOE | 1^st^ phalanx distalis |
